# RedoxiBase: A database for ROS homeostasis regulated proteins

**DOI:** 10.1016/j.redox.2019.101247

**Published:** 2019-06-06

**Authors:** Bruno Savelli, Qiang Li, Mark Webber, Achraf Mohamed Jemmat, Alexis Robitaille, Marcel Zamocky, Catherine Mathé, Christophe Dunand

**Affiliations:** a Laboratoire de Recherche en Sciences Végétales, Université de Toulouse, CNRS, UPS, 24 chemin de Borde Rouge, Auzeville, BP42617, 31326, Castanet-Tolosan, France; b Citrus Research Institute, Southwest University/Chinese Academy of Agricultural Sciences, Beibei, Chongqing, 400712, China; c Institute for Botany and Molecular Genetics, Bioeconomy Science Center, RWTH Aachen University, Aachen, Germany; d International Agency for Research on Cancer, Lyon, France; e Department of Molecular Evolution & Development University Vienna, Althanstrasse 14, A-1090, Vienna, Austria; f Laboratory of Phylogenomic Ecology, Institute of Molecular Biology, Slovak Academy of Sciences, Dubravska cesta 21, SK-84551, Bratislava, Slovakia

**Keywords:** Catalase, Peroxidases, Oxido-reductases, Multigenic family, ROS homeostasis

## Abstract

We present a new database, specifically devoted to ROS homeostasis regulated proteins. This database replaced our previous database, the PeroxiBase, which was focused only on various peroxidase families. The addition of 20 new protein families related with ROS homeostasis justifies the new name for this more complex and comprehensive database as RedoxiBase.

Besides enlarging the focus of the database, new analysis tools and functionalities have been developed and integrated through the web interface, with which the users can now directly access to orthologous sequences and see the chromosomal localization of sequences when available.

OrthoMCL tool, completed with a post-treatment process, provides precise predictions of orthologous gene groups for the sequences present in this database. In order to explore and analyse orthogroups results, taxonomic visualization of organisms containing sequence of a specific orthogroup as well as chromosomal distribution of the orthogroup with one or two organisms have been included.

## 1 Introduction

Reactive Oxygen Species (ROS) are represented by reactive molecules and free radicals derived from molecular oxygen: hydrogen peroxide, organic peroxides, superoxide, hydroxy radical, hydroxyl ion, singlet oxygen, and nitric oxide. They are produced at elevated concentrations during several essential biological processes such as respiration in most of living organisms, photosynthesis and photorespiration in chloroplastic organisms. They can also be released in a control manner during various developmental processes and stress responses. In particular, ROS can be produced as a part of innate immunity in Metazoans [1]. Although they can be deleterious, they are also necessary. To manage this ambivalent situation, each living being possesses a large battery of proteins which can produce or scavenge ROS in order to control their homeostasis. Among these proteins, haem or non-haem peroxidases were already centralized in a dedicated database namely the PeroxiBase [2].

In order to have a more integrative and phylogenomic overview on ROS-regulated proteins, new classes, families and superfamilies have been added to cover most of the proteins able to regulate ROS level. Then, the RedoxiBase, which includes all the data and the tools already present in the former PeroxiBase, was created. In the new database all living kingdoms are represented. The PeroxiBase served as a reference in the field of peroxidase families, the new enhanced version of this database should become a similar reference for all ROS regulation proteins. It is cross-referenced in UniProt [3] since 2006 and, more recently, in the Arabidopsis database TAIR [4].

Several databases centralize entries of all (InterPro [5]) or particular protein families (PLantCAZyme [6], CAZy [7], MEROPS [8], ThYme [9] and CaspBase, a curated database dedicated to the caspase family [10], or specific to a species such as GFDP which includes 6551 genes of poplar from 145 families [11]. Regarding the oxidase families, two independent databases are currently present in the web. Namely, PREX [12] is dedicated to only one type of non-haem peroxidases and fPOXDB [13] a fungal-specific database. They both bring structural and sequence information complementary to those found in our previous database PeroxiBase but they are merely devoted to subfamily assignment. Lastly, the antioxidant protein database AOD [14], was developed to understand the biological function of important antioxidant proteins but is was not maintained anymore.

Despite these different repositories, the (updated) RedoxiBase is still unique, since it is the only specialized collection of public sequences deduced from expert annotations with manual curation leading to re-annotation. Indeed, whole automatic genome annotation generates numbers of errors, notably with gene merging, splicing problems or tandem duplications [15]. These problems are exacerbated in the case of multigenic families like most proteins already included in our database. The guarantee of a high-quality sequence input is a prerequisite for performing reliable analyses, especially phylogeny. Efforts to provide only expert annotation derived sequences, in opposition to automated ones, exist elsewhere, but are still rather marginal.

Since its creation in 2004, the PeroxiBase has been a very active database with new sequences and new organisms daily added together with constant update of the interface with new tools and functionalities. Then, the RedoxiBase will take advantage of this existing dynamics to go further and pursue increase of available contents and features. Despite the explosion of genomic projects producing huge amounts of novel sequences that remain unexploited [16], the database will keep its initial interest to centralize high quality annotation for peroxidases and ROS-related proteins whereas it has only slightly evolved for semi-automatic annotation.

## 2 Description of tools and functions

### 2.1 Data available for each entry and tools

In April 2019, the database contains more than 15 000 sequences distributed over 2599 organisms. This brings an important biodiversity aspect and can grow further with availability of genomes from novel organisms. In addition to protein, cDNA, CDS, genomic, 2000 bp upstream and downstream sequences, the gene structure information (intron/exon structure), in Genbank format, is displayed along with a schematic representation.

The main challenge concerning large multigenic families is to obtain a comprehensive and reliable image of their evolution. To help establishing an evolutionary scenario, our interface provides many tools either to analyse the database entries or to compare them with input sequences. A regular BLAST including usual options (such as the nature of query and subject sequences and the choice of organism(s)) allows the users to search for sequences similar to their query in the database. Peroxiscan is a tool that provides the user with a prediction of a particular family or superfamily after testing the query sequence against pre-defined specific profiles [17]. CIWOG [18] and GECA [19] are tools that search for common introns in genes families based on intron position and protein sequence similarity around it. They return a graphical representation and comparison of several gene structures and highlight the conservation between sequences. The visualization of the alternative splicing, common in Metazoans, need to be developed. For multiple alignments, ClustalW and MAFFT are available directly online following multicriteria or BLAST searches, and a connection to the French phylogeny web site (http://www.phylogeny.fr) allows for further phylogenetic analysis. *Cis*-regulatory element analysis can be further performed with upstream and downstream sequences using PLACE [20] and MEME [21]. In addition, two major tools have been included for evolutionary and comparative genomic analyses and are described below.

### 2.2 New tool for evolutionary analysis: orthogroup

An orthogroup is defined as a group of peroxidases or ROS-related proteins that share a common ancestor. They are therefore either orthologs or paralogs. To perform clustering analysis and visualization, a specific pipeline, thereafter called ortho-pipeline, has been developed. This pipeline is based on OrthoMCL [22] and includes a post-treatment to reduce the false positives and negatives usually obtained with OrthoMCL. The originality and the relevance of our ortho-pipeline is to provide orthogroup classification even for partial sequences, based on sequence similarities.

**Fig. 1:**
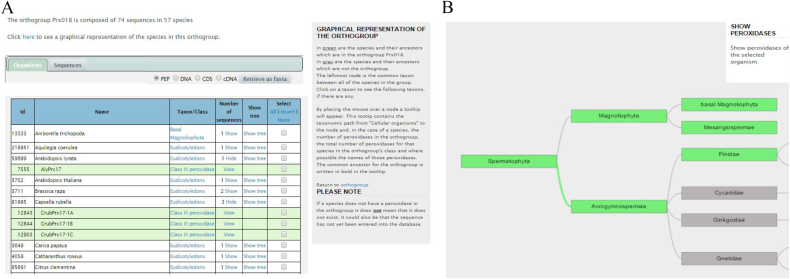
**Orthogroup pipeline results**. A. List of the organisms containing sequences belonging to the selected orthogroup. B. Visualization of the taxonomic distribution within an othogroup. Green boxes stand for organisms containing sequences belonging to the selected orthogroup. Gray boxes stand for organisms lacking sequences belonging to the selected orthogroup. (For interpretation of the references to colour in this figure legend, the reader is referred to the Web version of this article.)

Few new pages (Fig. 1A) were created on the web interface in order to visualize and analyse the taxonomic distribution of the orthogroups within different organisms. Graphical representation (Fig. 1B) of the orthogroup is available directly from one entry or from the tab “Browse the database by orthogroup” and “Analysis from input/Orthogroup search”. The green displayed the species and their ancestors, which possess sequences from the visualized orthogroup, while gray showed species that do not have sequences from the visualized orthogroup. The lack of sequence inside a visualized orthogroup can result from the absence of data or to the loss of sequence in a given species.

### 2.3 New tools for comparative genomics: Circos and chromodraw

**Fig. 2:**
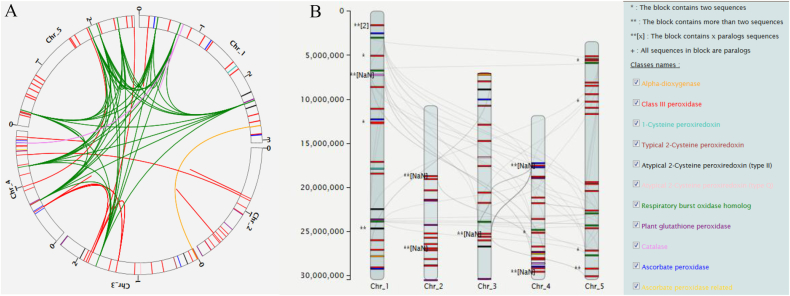
**Orthogroup pipeline visualization within one species**. A. Circos-like visualization. B. Chromosome Map visualization. Sequences belonging to the same orthogroup are linked. Each class is represented with one colour. Chromosome and gene loci on chromosomes are on scale. (For interpretation of the references to colour in this figure legend, the reader is referred to the Web version of this article.)

**Fig. 3:**
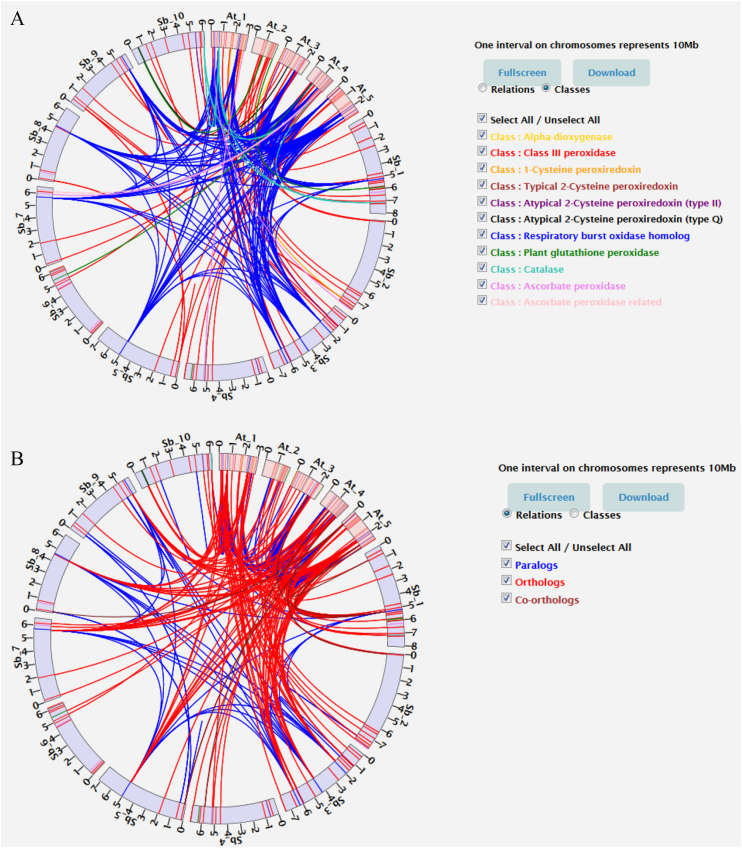
**Orthogroup pipeline visualization between two species**. A. Circos-like visualization of relation based on class belonging. B. Circos-like visualization of orthology/paralogy relations. Each class is represented with one colour. Chromosome and gene loci on chromosomes are on scale. (For interpretation of the references to colour in this figure legend, the reader is referred to the Web version of this article.)

As we are convinced that the information resulting from the orthoMCL-pipeline can play a major role to elucidate evolutionary history, an additional pipeline with chromosomal localization was developed: Circos-like visualization [23] and Chromosome Map (mapchart like [24]), allowing large scale genomic analysis, have been included. Standardised name for each chromosome, the location of each peroxidase or ROS-related protein on their respective chromosome (if available) and the paralogy/orthology relathionship obtained from OrthMCL pipeline were included in the final output (Fig. 2, Fig. 3).

### 2.4 New web interface and new code

As described above, the availability of a set of tools – some developed by our team - directly executable through the database website, facilitates evolutionary analysis. In addition, to improve the management of the database, as well as the speed of script execution and the database querying, the web application has been implemented in an open-source PHP framework (Codeigniter). This framework uses the Model-View-Controller concept and allows faster development, best security, better maintenance of the code and a reusability of applications developed in the laboratory with the same framework. Since 2008, the database is hosted by the GenoToul bioinformatics facility (http://bioinfo.genopole-toulouse.prd.fr). Recently, a new powerful computing cluster is available and can be used for local phylogenetic and clustering analysis.

## 3 Discussion and future prospects

With the accumulation of available genomes, the number of sequences included in the database was largely increased (from 6026 in 2008 [17] to 10710 in 2012 [2] and 15136 in 2019). Although, the numbers of organisms within each kingdom are in the same range, the RedoxiBase (formerly PeroxiBase) is still mainly composed of sequences originated from Viridiplantae (64%) and from fungi (22%). This is mainly due to the larger size of the red-ox proteins families found in plants and fungi which are subjected to large duplication events. Then, a particular effort needs to be done to increase the representation of ROS-related proteins from other kingdoms (mainly Protista and Animalia) and within them from exotic and poorly represented organisms. Special attention must be paid to genes from those species threatened with global extinction as reported recently by IPBES (Intergovernmental Science-Policy Platform on Biodiversity and Ecosystem Services Paris 2019). Regularly updating RedoxiBase with manually annotated sequences will allow to perform robust evolutionary analyses also for concatenated sequences.

The quality of the annotation, which is our main concern since the creation of the database, has been maintained, but manual annotation does not allow an efficient coverage of all the available sequences. The semi-automatic protocols developed will facilitate the upload of peroxidase-encoding sequences from already annotated proteomes while maintaining our high-quality standard. In addition, the annotation procedure relying on Scipio which has already demonstrated its effectiveness for gene prediction based on homology with closely related already annotated organisms [25], will be improved. Indeed, a new strategy that will take advantage of our specific profiles defined with controlled batches of sequences need to be developed for the prediction in more divergent genomes.

Many red-ox proteins families included in the RedoxiBase belong to multigenic families and result from tandem, segmental and chromosomal duplication events, which complicates global phylogenetic analysis and the understanding of their evolutionary history. The visualization of inter- or intra-species sequence orthogroup belonging and their chromosomal localization is very helpful in this context. This requires the availability of genomic localization for larger number of organisms. In addition, we have recently developed ExpressWeb, an online tool to perform gene clustering using personal or selected expressed value sets in order to construct co-expression gene networks [26]. ExpressWeb is available directly from the RedoxiBase and a current priority is to set up a pipeline to load publicly available expression data in order to perform expression clustering with our favorite genes.
